# Human Coding Synonymous Single Nucleotide Polymorphisms at Ramp Regions of mRNA Translation

**DOI:** 10.1371/journal.pone.0059706

**Published:** 2013-03-19

**Authors:** Quan Li, Hui-Qi Qu

**Affiliations:** 1 Endocrine Genetics Lab, The McGill University Health Center (Montreal Children's Hospital), Montréal, Québec, Canada; 2 Division of Epidemiology, Human Genetics and Environmental Sciences, The University of Texas School of Public Health, Houston, Texas, United States of America; New Jersey Institute of Technology, United States of America

## Abstract

According to the ramp model of mRNA translation, the first 50 codons favor rare codons and have slower speed of translation. This study aims to detect translational selection on coding synonymous single nucleotide polymorphisms (sSNP) to support the ramp theory. We investigated fourfold degenerate site (FFDS) sSNPs with A↔G or C↔T substitutions in human genome for distribution bias of synonymous codons (SC), grouped by CpG or non-CpG sites. Distribution bias of sSNPs between the 3^rd^ ∼50^th^ codons and the 51^st^ ∼ remainder codons at non-CpG sites were observed. In the 3^rd^ ∼50^th^ codons, G→A sSNPs at non-CpG sites are favored than A→G sSNPs [*P* = 2.89×10^−3^], and C→T at non-CpG sites are favored than T→C sSNPs [*P* = 8.50×10^−3^]. The favored direction of SC usage change is from more frequent SCs to less frequent SCs. The distribution bias is more obvious in synonymous substitutions CG(G→A), AC(C→T), and CT(C→T). The distribution bias of sSNPs in human genome, i.e. frequent SCs to less frequent SCs is favored in the 3^rd^ ∼50^th^ codons, indicates translational selection on sSNPs in the ramp regions of mRNA templates.

## Introduction

Synonymous DNA variations may affect mRNA function through the change of mRNA secondary structure, mRNA stability, synonymous codon (SC) usage, or co-translational protein folding [1]–[4]. With empirical evidence, synonymous single nucleotide polymorphisms (sSNP) in the *COMT* gene (encoding Catechol-O-Methyltransferase) may modulate pain sensitivity through the effect on mRNA secondary structure and efficiency of protein expression [5]–[7]. Examples of associations of sSNPs and human complex traits like the *COMT* sSNPs in pain sensitivity are rare. Most probably, although not functionally neutral, the functional effects of sSNPs are largely minor, while the minor effects are not readily identifiable by traditional genetic association study. SC usage bias is a widespread phenomenon across biological species [8]. A sSNP changing codon usage may be expected to fine-tune translational efficiency based on the availability of rare tRNAs [9], [10]. According to the ramp model of mRNA translation, except the second codon, the first 50 codons of mRNAs tend to favor rarer codons and have slower speed of translation [10]–[12]. This “ramp” mechanism is important in determining translation efficiency, preventing ribosome congestion, and allowing proper co-translational folding of proteins [3]. Based on the ramp theory, human sSNPs at ramp regions may confront selection pressure because of their functional effect on codon usage. To identify the translational effect of an individual SNP is difficult. Instead, we tried to identify the overall selection effect on sSNPs in human genome in this study. We investigated the incidences of sSNPs in the 3^rd^∼50^th^ codons vs. those in the remainder codons after the 51^st^ codon.

## Methods

Fourfold degenerate site (FFDS, i.e. the four nucleotides A/C/G/T at this site encode the same amino acid) sSNPs with A↔G or C↔T substitutions in human genome were extracted from the NCBI dbSNP database build 134 (http://www.ncbi.nlm.nih.gov/projects/SNP/). Altogether, 39,276 sSNPs in 12,568 genes were collected. All SNP alleles were corresponding to the nucleotides in coding sequences. Among these FFDS sSNPs, 20,122 were A↔G sSNPs, and 19,154 were C↔T sSNPs. Of the 20,122 A↔G FFDS sSNPs, 43 at second codons of coding regions were removed from further analysis; of 19,154 C↔T sSNPs, 25 at second codons were removed from further analysis. The FFDS sSNPs were annotated as N_1_→N_2_, while N_1_ represents the ancestral allele and N_2_ represents the variant allele. Ancestral alleles of sSNPs were inferred by human-chimpanzee genomic alignment according to the SeattleSeq Annotation 134 (http://snp.gs.washington.edu/SeattleSeqAnnotation134/index.jsp). All sSNPs were differentiated by CpG sites *versus* non-CpG sites, while a CpG site has the pattern of YpG or CpR (Y represents C↔T substitution, and R represents A↔G substitutions).

## Results

**Figure 1:**
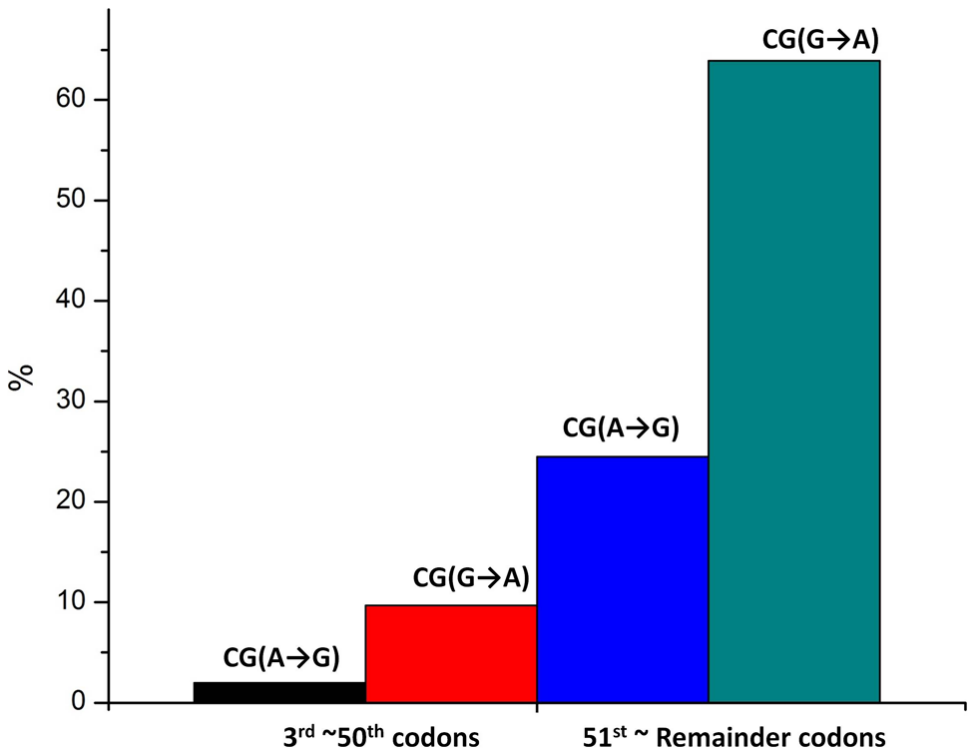
The distribution bias of CG(G→A) and CG(A→G) at the ramp regions. The ratio of CG(G→A)/CG(A→G) at the ramp regions is larger than that at the reminder coding regions (*P* = 0.040). CG(A↔G) synonymous substitutions are all at non-CpG sites.

**Figure 2:**
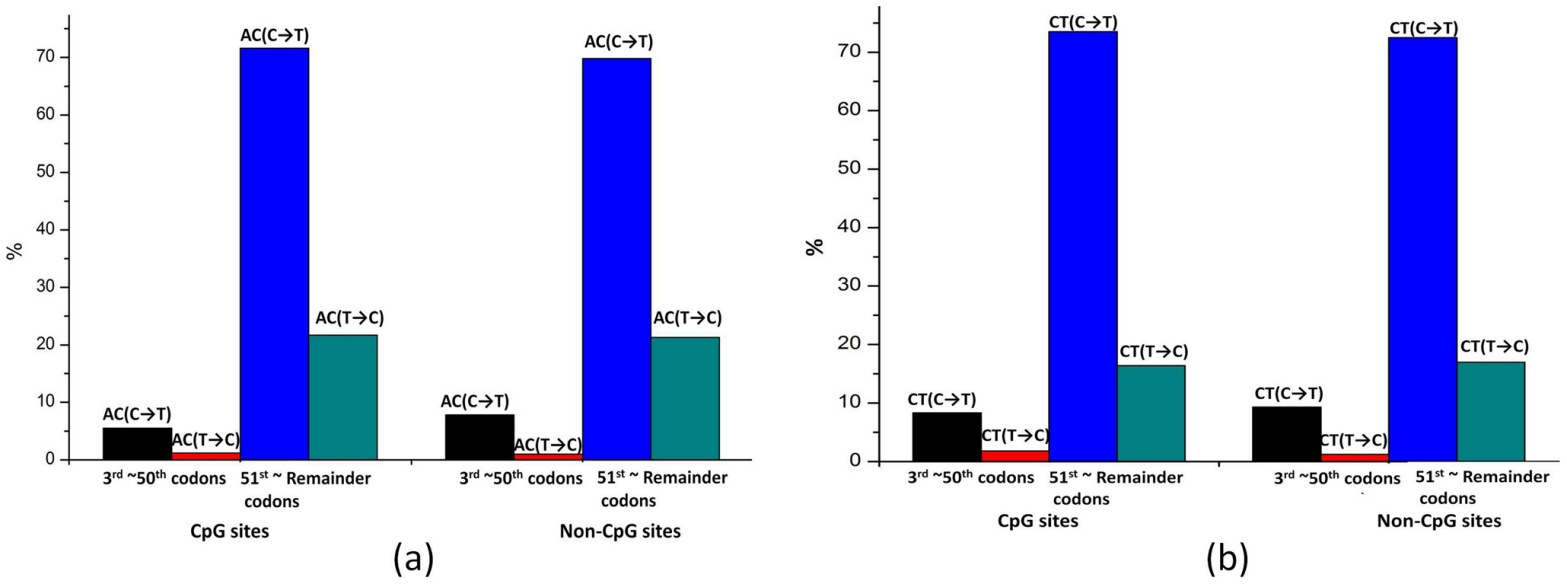
The distribution bias of (C→T) and (T→C) at non-CpG sites of the ramp regions. (a) The ratio of AC(C→T)/AC(T→C) at non-CpG sites of the ramp regions is larger than that at the reminder coding regions(*P* = 0.006). (b) The ratio of CT(C→T)/CT(T→C) at non-CpG sites of the ramp regions is larger than that at the reminder coding regions (*P* = 0.029).

Our results showed that the fraction of FFDS sSNPs is significantly lower in the ramp (the 3^rd^ ∼50^th^ codons) than the rest regions (after the 50^th^ codon) [0.23% *vs*. 0.32%, odds ratio OR (95% confidence interval CI)  = 0.708 (0.684, 0.734), *P* = 1.60×10^−81^), corrected by the FFDS codon usages calculated by the European Molecular Biology Laboratory (EMBL) Human CDSs (Coding sequences) Release 115 (ftp://ftp.ebi.ac.uk/pub/databases/embl/cds/). We identified significant distribution bias of sSNPs between the 3^rd^ ∼50^th^ codons and the 51^st^ ∼ remainder codons at non-CpG sites (Table 1). This distribution bias at non-CpG sites is consistent with our previous study on the asymmetry pattern of complementary sSNPs at FFDS, which was seen in non-CpG sSNPs only, but not sSNPs at CpG sites. This context-specific distribution bias is related to lower mutation rates and longer periods of evolutionary selection at non-CpG sites [13]. In the 3^rd^ ∼50^th^ codons, G→A sSNPs are favored than A→G sSNPs at non-CpG sites [OR (95% CI)  = 1.353 (1.108, 1.652)], and C→T sSNPs are favored than T→C sSNPs at non-CpG sites [OR (95% CI)  = 1.272(1.063, 1.523)]. In both cases of G→A and C→T, the favored direction of SC usage is the change from more frequent SCs to less frequent SCs. The reference data of human codon usage (Table S1) was calculated by the EMBL human coding sequences (CDS) data release 115 (ftp://ftp.ebi.ac.uk/pub/databases/embl/cds/). By further investigation, our study disclosed that the G→A bias was mainly seen in synonymous substitution CG(G→A) at non-CpG sites [OR (95% CI)  = 1.861(1.020, 3.395)] (Table 2, Figure 1); the C→T bias was mainly seen in AC(C→T) [OR (95% CI)  = 2.275 (1.255, 4.124)] and CT(C→T) [OR (95% CI)  = 1.780 (1.053, 3.010)] at non-CpG sites (Table 3, Figure 2). In all these three types of biased synonymous substitutions [i.e. CG(G→A), AC(C→T), and CT(C→T)], the favored change at the ramp region is from more frequent SCs to less frequent SCs.

**Table 1:** A↔G and C↔T fourfold degenerate site sSNPs.

Substitution type	A→G n(%)	G→A n(%)	C→T n(%)	T→C n(%)	Total (count)
**NonCpG site**					
3rd ∼50th codons	137(9.3%)	453(30.7%)	727(49.3%)	158(10.7%)	1475
51st ∼ Remainder codons	1632(11.6%)	3988(28.5%)	6575(46.9%)	1818(13.0%)	14013
3rd ∼50th codons *vs*. remainders a	p = 2.89×10^−3^ **		p = 8.50×10^−3^ **		
**CpG site**					
3rd ∼50th codons	283(15.2%)	760(40.9%)	642(34.6%)	173(9.3%)	1858
Remainder codons	3614(16.5%)	9212(42.1%)	7046(32.2%)	1990(9.1%)	21862
3rd ∼50th codons *vs*. remainders a	p = 0.471		p = 0.599		

a χ^2^ test of the difference of substitution direction between the first 50 codons and the remainder codons; * *P*<0.05; **P<0.01.

Significant distribution bias of sSNPs between the 3^rd^ ∼50^th^ codons and the 51^st^ ∼ remainder codons was identified at non-CpG sites.

**Table 2:** A↔G fourfold degenerate site sSNPs.

First two codon positions	CpG site (yes or no)	3^rd^ ∼50^th^ codons		51^st^ ∼ Remainder codons		3^rd^ ∼50^th^ codons *vs.* remainders (*P* value) a
		A→G n(%)	G→A n(%)	A→G n(%)	G→A n(%)	
AC	Yes	68(1.8%)	164(4.4%)	1021(27.1%)	2508(66.7%)	0.902
CC	Yes	84(2.1%)	226(5.7%)	1039(26.2%)	2621(66%)	0.628
CG	No	14(2%)	68(9.7%)	172(24.5%)	449(63.9%)	0.040*
CT	No	50(2.2%)	171(7.4%)	601(26%)	1491(64.5%)	0.055
GC	Yes	72(2%)	246(6.8%)	907(25.2%)	2369(65.9%)	0.054
GG	No	40(2.6%)	96(6.3%)	499(32.8%)	887(58.3%)	0.125
GT	No	33(2%)	118(7.1%)	360(21.5%)	1161(69.4%)	0.616
TC	Yes	59(2.3%)	124(4.9%)	647(25.4%)	1714(67.4%)	0.159

a χ^2^ test of the difference of substitution direction between the first 50 codons and the remainder codons; *Uncorrected *P*<0.05. By Bonferroni correction for multiple comparisons, the threshold for statistical significance is *P*<0.00625.

The G→A bias was mainly explained by the CG(G→A) substitution at non-CpG sites.

**Table 3:** C↔T fourfold degenerate site sSNPs.

First two codon positions	CpG site (yes or no)	3^rd^ ∼50^th^ codons		51^st^ ∼ Remainder codons		3^rd^ ∼50^th^ codons *vs.* remainders (*P* value) a
		C→T n(%)	T→C n(%)	C→T n(%)	T→C n(%)	
AC	Yes	79(5.5%)	17(1.2%)	1027(71.6%)	311(21.7%)	0.212
AC	No	97(7.8%)	13(1%)	866(69.8%)	264(21.3%)	0.006**
CC	Yes	106(6%)	31(1.8%)	1249(70.8%)	377(21.4%)	0.882
CC	No	81(6.2%)	26(2%)	906(69.1%)	298(22.7%)	0.917
CG	Yes	42(7.1%)	8(1.4%)	437(73.9%)	104(17.6%)	0.578
CG	No	48(9.6%)	6(1.2%)	377(75.7%)	67(13.5%)	0.435
CT	Yes	69(8.3%)	15(1.8%)	613(73.5%)	137(16.4%)	0.927
CT	No	129(9.3%)	17(1.2%)	1006(72.5%)	236(17%)	0.029*
GC	Yes	127(6%)	35(1.7%)	1514(71.9%)	430(20.4%)	0.879
GC	No	126(7.8%)	27(1.7%)	1165(72.2%)	296(18.3%)	0.442
GG	Yes	116(8.8%)	25(1.9%)	958(72.7%)	219(16.6%)	0.800
GG	No	103(8.8%)	20(1.7%)	834(71.2%)	215(18.3%)	0.267
GT	Yes	33(5.7%)	13(2.2%)	408(70.2%)	127(21.9%)	0.491
GT	No	40(5%)	14(1.8%)	593(74.7%)	147(18.5%)	0.285
TC	Yes	70(5.7%)	29(2.4%)	840(68.6%)	285(23.3%)	0.387
TC	No	103(8.2%)	35(2.8%)	828(65.7%)	295(23.4%)	0.819

a χ^2^ test of the difference of substitution direction between the first 50 codons and the remainder codons; *Uncorrected *P*<0.05; **Uncorrected *P*<0.01. By Bonferroni correction for multiple comparisons, the threshold for statistical significance is *P*<0.003125.

The C→T bias was mainly explained by the AC(C→T) and CT(C→T) substitutions at non-CpG sites.

To further characterize the distribution bias of FFDS sSNPs, we examined distributions of FFDS sSNPs stepwisely by comparing the 3^rd^∼n^th^ (n = 20, 21, …,60) codons vs. the remainder codons (Table S2). The overall C→T bias at non-CpG sites was most significant in the first 46 codons. The codon-specific AC(C→T) bias at non-CpG sites was most significant in the first 50 codons, and the codon-specific CT (C→T) bias at non-CpG sites was most significant in the first 45 codons. The overall G→A bias at non-CpG sites was most significant in the first 55 codons, and the codon-specific CG(G→A) bias at non-CpG sites was most significant in the first 39 codons. Therefore, the ramp region may not have a clear border in term of codon number. As a side note, the GG(G→A) bias at non-CpG sites also showed nominal significance in the first 57 codons (*P* = 0.021), and the CT(G→A) bias at non-CpG sites was nominal significant in the first 46 codons (*P* = 0.026). The change of codon usage of CT(G→A) has also the direction from more frequent SC to less frequent SC. The change of codon usage of GG(G→A) is unobvious. One exception is the statistical significance of GC(G→A) bias (*P* = 1.85×10^−3^) in the first 25 codons. These GC(G→A)s have the codon usage change from less frequent GCG to more frequent GCA. The GC(G→A) bias disappeared when more codons (≥45 codons) in the ramp region are considered.

## Discussion

Our previous study showed genome-wide discrepancy of human sSNPs between two complementary DNA strands, and suggested widespread selective pressure due to functional effects of sSNPs related to gene transcription [13]. The asymmetry pattern of complementary sSNPs in human genome may be related to transcription-coupled mutation and repair [13]. In this study, we identified another type of distribution bias of sSNPs in human genome related to mRNA translation. Biased directions of SC substitutions between the 3^rd^ ∼50^th^ codons and the 51^st^ ∼ remainder codons at non-CpG sites were observed. In the 3^rd^ ∼50^th^ codons, G→A sSNPs at non-CpG sites are favored than A→G sSNPs, and C→T at non-CpG sites are favored than T→C sSNPs. In both cases, the change from more frequent SCs to less frequent SCs is favored in the 3^rd^ ∼50^th^ codons over the remainder codons. This finding is supportive to the ramp model of SC uage in mRNA translation [10], [11]. The change from more frequent SCs to less frequent SCs may enhance the function of ramp regions to prevent subsequent ribosome congestion and improve the efficiency of protein synthesis. On the other hand, if a synonymous substitution has the change of a less frequent SC to a more frequent SC, it may impair ramp function and cause ribosomal traffic jams during protein synthesis. The potential deleterious effect of these sSNPs may be subjected to larger evolutionary selection pressure, and tend to be removed by purifying selection.

By investigating 13,798 common sSNPs genotyped by the HapMap3 project, Waldman et al. demonstrated evolutionary selection for translation efficiency on sSNPs [14]. By investigating all human sSNPs, our study identified the obvious bias in the ramp region for synonymous substitutions CG(G→A), AC(C→T), and CT(C→T), indicating codon-specific effect on gene translation efficiency. As a limitation of this study, the specific SC changes that we identified didn't reach the significance level after correction of multiple testing by Bonferroni correction, which warrants for further study. On the other hand, empirically, codon-specific translation efficiency has been observed in model organisms, e.g. the strongly inhibitory effect of the CGA codon in yeast [15]. The intriguing exception of the GC(G→A) bias may suggest that the hypermutable GCG through methylation-induced deamination of 5-methyl cytosine on the antisense strand [16] meets less negative selection in the first half of the ramp region, but stronger negative selection in the second half of the ramp region which compensates the GC(G→A) bias in the first half of the ramp region. The lack of negative selection on GC(G→A) in the first 25 codons may suggest a functional heterogeneity of the ramp region, which warrants further study. In addition, Tuller et al. recently highlighted that stronger mRNA folding may also be involved in the ramp function [17]. Different effect of these SCs on mRNA secondary structure is an interesting issue deserving further inquiry.

## Supporting Information

Table S1 **Human codon usage calculated by the EMBL human coding sequences (CDS) data release 115.**
(DOC)

Table S2 **Stepwise analysis of the distribution bias of FFDS sSNPs.** FFDS sSNPs were analyzed in different size windows of the initial segments of coding sequences.
(XLS)
