# Supplementary material for: Human Coding Synonymous Single Nucleotide Polymorphisms at Ramp Regions of mRNA Translation
Source: PLoS One. 2013 Mar 19;8(3):e59706. doi: 10.1371/journal.pone.0059706 (PMC3602041; doi:10.1371/journal.pone.0059706)
Supplement: Table S1 — Human codon usage calculated by the EMBL human coding sequences (CDS) data release 115. (DOC) [file pone.0059706.s001.doc]

**Supplementary Table 1 Human codon usage calculated by the EMBL human coding sequences (CDS) data release 115** (<ftp://ftp.ebi.ac.uk/pub/databases/embl/cds/>).

| **Codon** | **Frequency (‰)** | **Count** |
| --- | --- | --- |
| AAA | 25.6 | 679,486 |
| AAC | 18.4 | 488,951 |
| AAG | 31.8 | 845,395 |
| AAU | 17.3 | 460,042 |
| ACA | 15.7 | 415,572 |
| ACC | 18.1 | 479,814 |
| ACG | 5.9 | 156,094 |
| ACU | 13.6 | 361,114 |
| AGA | 12.7 | 336,371 |
| AGC | 19.6 | 519,302 |
| AGG | 12.2 | 324,120 |
| AGU | 13.0 | 344,322 |
| AUA | 7.7 | 203,670 |
| AUC | 19.6 | 519,319 |
| AUG | 21.8 | 580,059 |
| AUU | 16.2 | 429,170 |
| CAA | 13.0 | 346,217 |
| CAC | 14.9 | 394,818 |
| CAG | 34.9 | 925,981 |
| CAU | 11.3 | 299,122 |
| CCA | 18.1 | 480,585 |
| CCC | 19.7 | 522,448 |
| CCG | 6.8 | 181,577 |
| CCU | 18.5 | 491,641 |
| CGA | 6.3 | 167,736 |
| CGC | 9.8 | 260,697 |
| CGG | 11.4 | 301,886 |
| CGU | 4.6 | 123,053 |
| CUA | 7.3 | 194,692 |
| CUC | 18.8 | 499,006 |
| CUG | 38.1 | 1,012,277 |
| CUU | 13.8 | 366,078 |
| GAA | 31.1 | 825,475 |
| GAC | 24.6 | 652,321 |
| GAG | 39.5 | 1,050,048 |
| GAU | 22.6 | 600,847 |
| GCA | 16.6 | 441,566 |
| GCC | 27.1 | 718,598 |
| GCG | 6.9 | 183,769 |
| GCU | 18.9 | 502,072 |
| GGA | 17.1 | 454,613 |
| GGC | 21.4 | 568,392 |
| GGG | 16.2 | 430,801 |
| GGU | 11.0 | 291,559 |
| GUA | 7.4 | 196,565 |
| GUC | 13.9 | 367,982 |
| GUG | 27.0 | 715,707 |
| GUU | 11.4 | 303,507 |
| UAA | 0.6 | 15,165 |
| UAC | 14.1 | 373,099 |
| UAG | 0.4 | 11,871 |
| UAU | 11.9 | 317,018 |
| UCA | 13.0 | 346,133 |
| UCC | 17.4 | 463,041 |
| UCG | 4.3 | 114,942 |
| UCU | 15.8 | 420,070 |
| UGA | 1.0 | 26,974 |
| UGC | 11.8 | 314,277 |
| UGG | 12.6 | 334,130 |
| UGU | 10.5 | 278,659 |
| UUA | 8.2 | 217,041 |
| UUC | 18.8 | 498,178 |
| UUG | 13.2 | 350,223 |
| UUU | 17.2 | 457,760 |
